# Selective CAR T cell–mediated B cell depletion suppresses IFN signature in SLE

**DOI:** 10.1172/jci.insight.179433

**Published:** 2024-05-09

**Authors:** Artur Wilhelm, David Chambers, Fabian Müller, Aline Bozec, Ricardo Grieshaber-Bouyer, Thomas Winkler, Dimitrios Mougiakakos, Andreas Mackensen, Georg Schett, Gerhard Krönke

**Affiliations:** 1 Department of Internal Medicine 3 - Rheumatology and Immunology, FAU Erlangen-Nürnberg and Universitätsklinikum Erlangen, Erlangen, Germany.; 2 Deutsches Zentrum Immuntherapie, Universitätsklinikum Erlangen, Friedrich-Alexander University (FAU) Erlangen - Nürnberg, Erlangen, Germany.; 3 Department of Internal Medicine 5 - Hematology and Oncology, FAU Erlangen-Nürnberg and Universitätsklinikum Erlangen, Erlangen, Germany.; 4 Department of Genetics, Friedrich-Alexander University (FAU) Erlangen - Nürnberg, Erlangen, Germany.; 5 Department of Hematology and Oncology, Otto-von-Guericke University Magdeburg, Magdeburg, Germany.; 6 Department of Rheumatology and Clinical Immunology, Charité, Unversitätsmedizin Berlin, Berlin, Germany.

**Keywords:** Autoimmunity, Immunology, Autoimmune diseases, Bioinformatics

## Abstract

Applying advanced molecular profiling together with highly specific targeted therapies offers the possibility to better dissect the mechanisms underlying immune-mediated inflammatory diseases such as systemic lupus erythematosus (SLE) in humans. Here we apply a combination of single-cell RNA-Seq and T/B cell repertoire analysis to perform an in-depth characterization of molecular changes in the immune-signature upon CD19 CAR T cell–mediated depletion of B cells in patients with SLE. The resulting data sets not only confirm a selective CAR T cell–mediated reset of the B cell response but simultaneously reveal consequent changes in the transcriptional signature of monocyte and T cell subsets that respond with a profound reduction in type I IFN signaling. Our current data, thus, provide evidence for a causal relationship between the B cell response and the increased IFN signature observed in SLE and additionally demonstrate the usefulness of combining targeted therapies and analytic approaches to decipher molecular mechanisms of immune-mediated inflammatory diseases in humans.

## Abstract 2

A combination of single cell RNA-sequencing and T/B cell repertoire analysis reveal molecular changes in the immune-signature upon CD19 CAR T cell-mediated depletion of B cells in patients with systemic lupus erythematosus.

## Introduction

Systemic lupus erythematosus (SLE) represents a prototypic autoimmune disease characterized by chronic inflammation and progressive inflammation-associated tissue damage of multiple organs including kidneys, joints, and skin (1). SLE is believed to result from a break in systemic immune tolerance, with patients with SLE displaying autoantibodies against ubiquitous nuclear antigens such as double-stranded DNA and histones (2). B cells seem to exert a pivotal role during the pathogenesis of SLE, where they are considered to act as precursors of autoantibody-producing plasmablasts and plasma cells and additionally provide the base of a pathogenic immunological memory in the form of autoreactive memory B cells that are able to sustain persistent autoimmunity (3).

Another hallmark of SLE is the presence of an increased type I IFN signature in the form of an enhanced expression of multiple IFN-inducible genes in peripheral blood mononuclear cells (PBMCs) indicating an additional key role of IFNs in the development of SLE (4–6). In accordance, a range of genetic disorders that result in increased type I IFN production or the treatment with recombinant type I IFNs themselves can trigger onset of an SLE-like disease pathology (7, 8). Blockade of the type I IFN receptor has consequently emerged as an effective therapy for a subset of patients with SLE (9).

Together, these findings have provoked important questions not only about the cellular sources and targets of type I IFNs but also about the triggers and the causative series of events that eventually foster IFN production and B cell activation, respectively, in patients with SLE.

Previous clinical trials in patients with SLE studying B cell depletion yielded discrepant results (10), which were partially due to the inefficient depletion of tissue B cells in response to therapeutic CD20 antibodies (11). Meanwhile, CD19 CAR T cells have emerged as a potentially novel therapeutic tool that has demonstrated superiority to antibody-mediated B cell depletion during the therapy of B cell lymphomas and B cell leukemias (12). Increasing evidence indicates that CD19 CAR T cell therapy also enables a profound depletion of B cells in patients with SLE and patients suffering from other types of B cell–mediated autoimmune disease, an approach that was able to facilitate a sustained and drug-free remission in such patients (13–16). Studying the molecular and cellular effects of a CD19 CAR T cell–mediated elimination of B cells in patients with SLE, thus, provides a unique opportunity to directly learn from precision medicine and better understand the contribution of B cells to the pathogenesis of SLE as part of a reverse translational approach.

In the current manuscript, we provide data on the molecular role of B cells in SLE that are based on single-cell RNA-Seq (scRNA-Seq) of PBMCs as well as a repertoire analysis of B cell and T cell receptors in patients with SLE before and after CD19 CAR T cell therapy. The insights derived from this analysis not only support a CD19 CAR T cell–mediated reset of the memory B cell compartment but also the parallel inhibition of the IFN signature in monocytes and T cells of patients with SLE. CD19 CAR T cell therapy as precision medicine approach in conjunction with deep molecular phenotyping, thus, provides support for the central role of autoreactive B cells during the pathogenesis of SLE as well as for the B cell–dependent increase in type I IFN signaling previously observed in PBMCs of patients with SLE.

## Results

We conducted a scRNA-Seq–based analysis of PBMCs derived from a cohort of patients with SLE (Supplemental Table 1; supplemental material available online with this article; https://doi.org/10.1172/jci.insight.179433DS1) before and after a therapy with CD19 CAR T cells. The analysis after CD19 CAR T cell treatment was performed at a stage when, after initial depletion, de novo B cells had already repopulated the peripheral blood. Numbers of peripheral blood B cells were monitored and B cell repopulation was observed in the individual patients between 9 and 28 weeks after CD19 CAR T cell treatment (Supplemental Table 2). This approach enabled us to study the global effect of a defined CD19 CAR T cell–mediated depletion of B cells on the immune status of patients with SLE and to consequently understand the precise contribution of B cell (auto-)reactivity to the molecular immune pathogenesis of SLE. Recent data show a profound clinical and serological response of these patients, who entered a drug-free remission with normalization of clinical disease activity parameters and showed a reduction or disappearance of autoantibodies despite the repopulation of B cells (16). These findings are suggestive of a deep reset of autoimmunity in response to CD19 CAR T cell therapy in this cohort of patients with SLE (14, 16).

**Figure 1:**
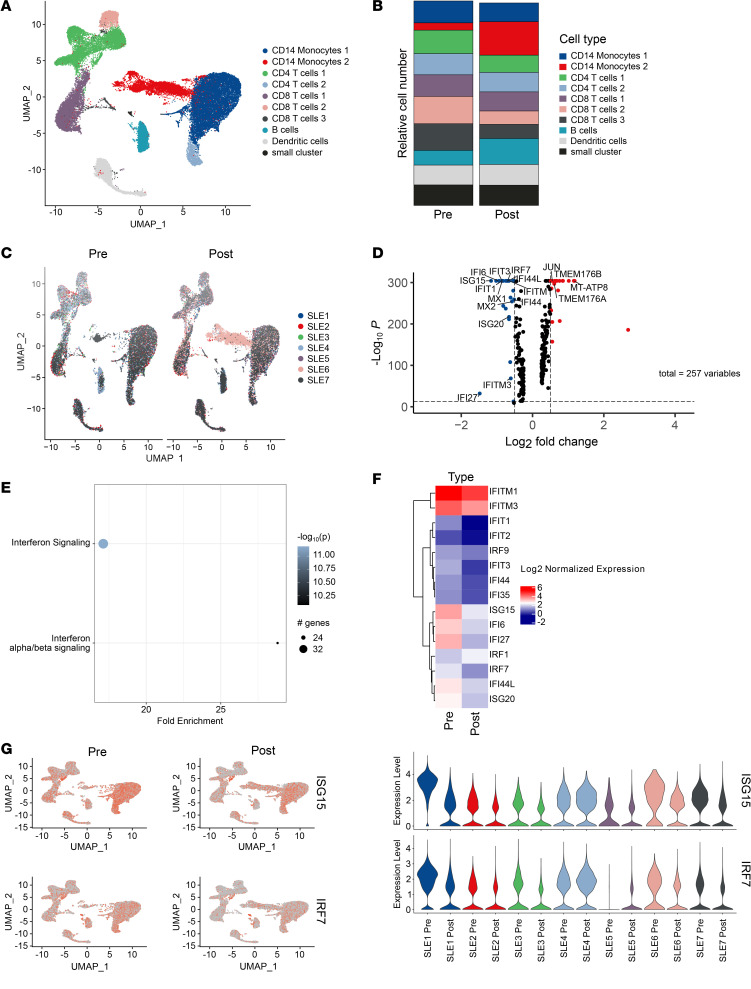
scRNA-Seq–based analysis of PBMCs of patients with SLE. (**A**–**G**) Single-cell RNA-Seq–based (scRNA-Seq–based) analysis of peripheral blood mononuclear cells (PBMCs) of patients with SLE (*n* = 7) before CD19 CAR T cell therapy and after early B cell repopulation. Single data sets from each patient were integrated using the Seurat pipeline into one data set containing approximately 40,700 PBMC. (**A**) Dimensional reduction of PBMCs revealing indicated UMAP clusters where cluster identities were determined through top cluster gene analysis and the Enrichr package. (**B** and **C**) Comparison of single-cell data sets from patients with SLE before anti-CD19 CAR T cell therapy and after early B cell reconstitution. (**D**) Differential gene expression analysis on the effect of anti-CD19 CAR T cell therapy. (**E**) Pathway enrichment analysis based on changes in gene expression of total PBMCs. The color intensity reflects the adjusted *P* value for each pathway. (**F**) IFN-associated genes are highlighted in a normalized heatmap representing mean gene expression of interferon related genes (*n* = 15 unique genes) before and after CD19 CAR T cell treatment. (**G**) Gene expression levels of *ISG15* and *IRF7* as representative IFN-induced genes are highlighted as a feature plot and in violin plots illustrating expression levels on the basis of individual patients.

Our current scRNA-Seq analysis shows that, apart from an initial depletion of B cells, CD19 CAR T cell treatment did not provoke major changes in the basic cellular composition of T cells and monocytes at a stage when de novo B cells had repopulated the peripheral blood (Figure 1, A–C). To understand enduring molecular consequences of a singular CD19 CAR T cell–mediated reset of the B cell response, we subsequently performed a pathway enrichment analysis (PEA) based on changes in gene expression of total PBMCs in response to therapy (Figure 1, D and E). Notably, IFN signaling in general, and type I IFN (IFN-α/β) signaling in particular, emerged as the major pathways that were decreased in response to CD19 CAR T cell treatment. We accordingly observed a significant reduction in the expression of a large set of IFN-induced genes such as *IRF7* or *ISG15* that, prior to CD19 CAR T cell therapy, were primarily expressed within monocyte and T cell subsets of this cohort of patients with SLE (Figure 1, F and G).

**Figure 2:**
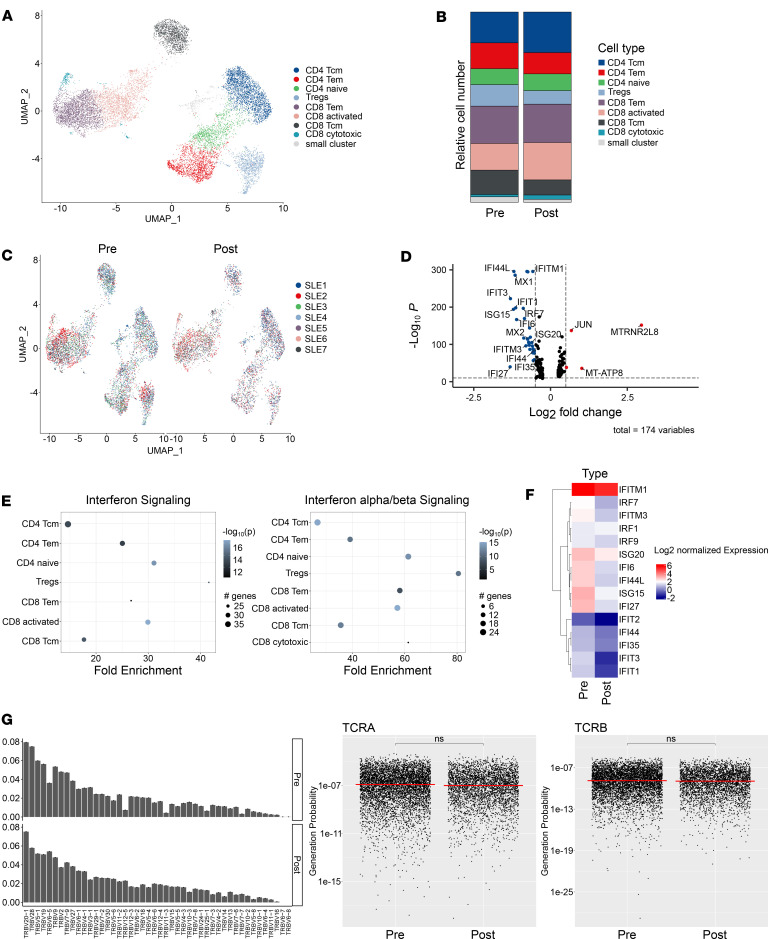
CD19 CAR T cell–mediated changes in T cell signature and TCR repertoire. (**A**–**C**) scRNA-Seq–based reclustering and visualization of CD4^+^ and CD8^+^ T cells before and after CD19 CAR T cell treatment using dimensionality reduction on a 2D scale in a UMAP plot. (**D** and **E**) Volcano plot and pathway enrichment analysis showing the effect of CD19 CAR T cell treatment on the differential gene expression. (**F**) Heatmap displaying changes of IFN-dependent genes upon CD19 CAR T cell treatment. (**G**) scRNA-Seq–based T cell receptor repertoire analysis showing V gene usage of TCR-β chains and probability of TCR chain generation based on calculation with the OLGA algorithm. Statistical analyses were performed using the median and 1-tailed *t* test with Benjamini-Hochberg correction.

A more detailed analysis of the molecular signature of individual T cell subsets within the peripheral blood did not reveal major changes in the frequency of CD4^+^ or CD8^+^ T cells, and they did not reveal major changes in the frequency of Tregs in response to CD19 CAR T cell therapy (Figure 2, A–C). Analysis of differential gene expression changes as well as PEAs, specifically performed within the T cell data set, again identified IFN signaling as the top regulated pathway and identified different IFN-induced genes that were downregulated in patients with SLE upon CD19 CAR T cell therapy in different T cell subsets (Figure 2, D–F). Repertoire analysis showed that CD19 CAR T cell therapy did not affect the T cell receptor (TCR) repertoire, with T cells maintaining a stable distribution in the expression of TCR-β chains before and after treatment (Figure 2G). We additionally performed an optimized likelihood estimate analysis to compute the generation probability of detected CDR3 sequences within the sequenced TCRs (Figure 2G). This approach showed that the calculated generation probabilities for both TCR-α and TCR-β chains did not differ before or after CD19 CAR T cell therapy, indicating that the T cell repertoire remained unaffected in response to this therapeutic approach. Among the data sets obtained after B cell reconstitution, we could not detect CAR T cells.

**Figure 3:**
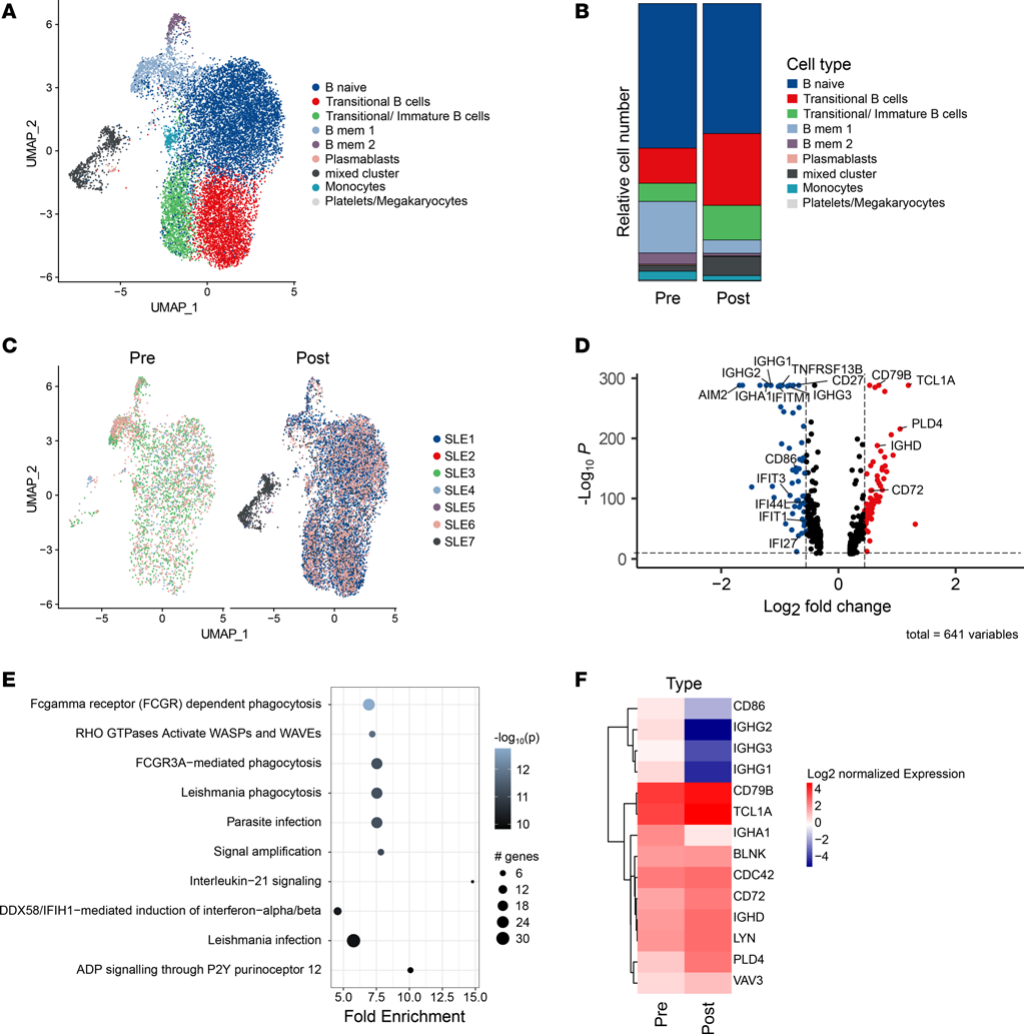
CD19 CAR T cell–mediated changes in B cell signature. (**A**–**F**) scRNA-Seq–based analysis of data sets generated from sorted B cells before and after CD19 CAR T cell therapy illustrated as grouped individual UMAP plots (**A** and **C**) and calculated relative cell number of indicated B cell subsets (**B**); differential gene expression (**D**), gene expression-based pathway analysis (**E**), and expression of BCR and FcγR signaling related genes (**F**) in scRNA-Seq data sets derived from isolated B cells before and after CD19 CAR T cell therapy.

Finally, we analyzed the scRNA-Seq–based gene expression profile and repertoire of B cells prior to and past CD19 CAR T cell treatment after B cell reconstitution. This approach showed a substantial reduction of memory B cells as well as a parallel expansion of transitional and transitional/immature B cells upon the CAR T cell–induced reset of the B cell compartment (Figure 3, A–C). In accordance with an early and/or intermediate developmental stage of reconstituted B cells, we observed an enhanced expression of genes such as *PLD4* in this cellular compartment when studying differential gene expression in B cells before and after CAR T cell therapy (Figure 3D). PEA accordingly showed that the pathways, which changed upon repopulation of B cells in response to CD19 CAR T cell treatment, were mostly associated with BCR and FcγR signaling (Figure 3, E and F).

**Figure 4:**
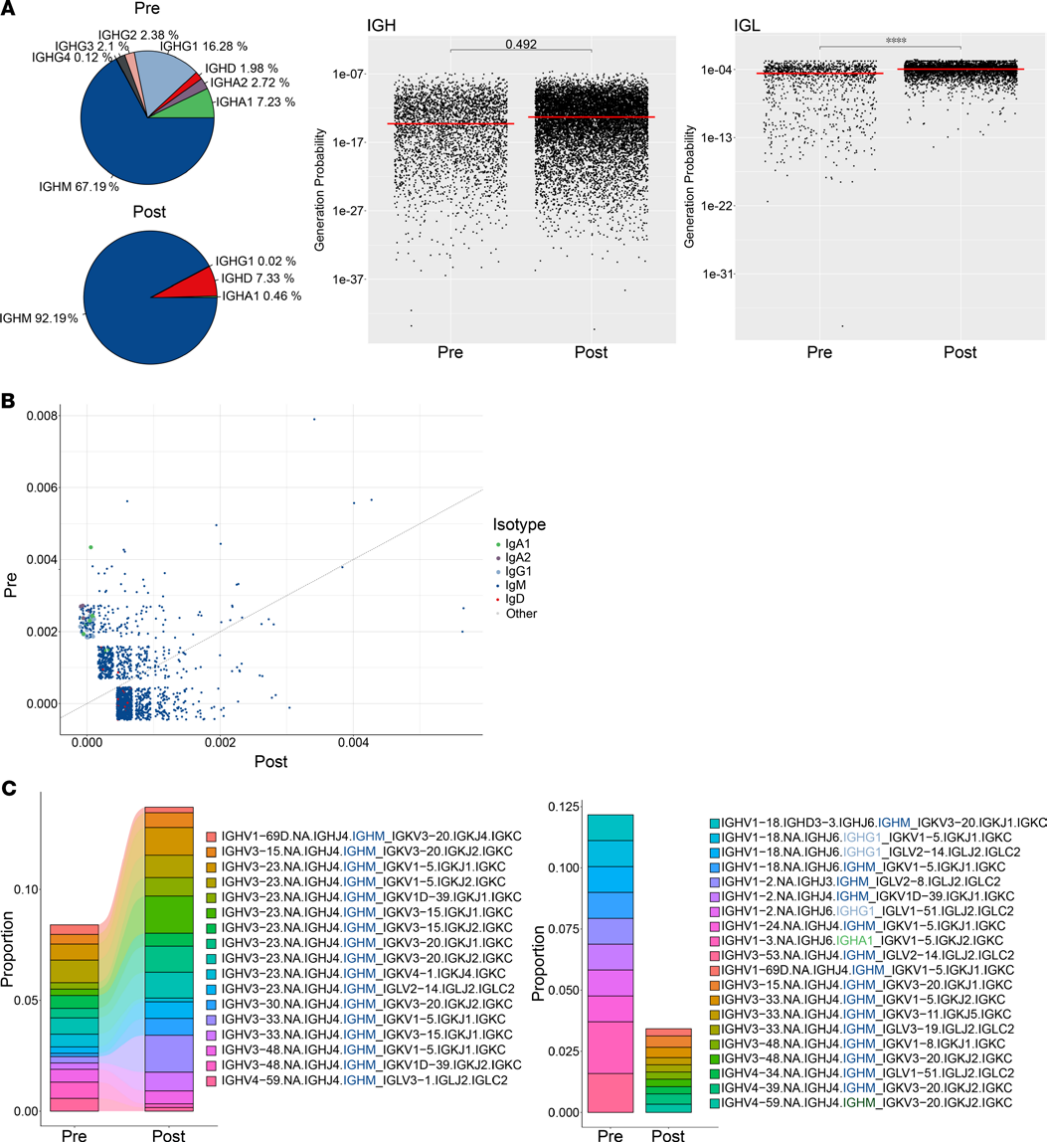
CD19 CAR T cell–mediated changes in B cell repertoire. (**A**) BCR repertoire analysis demonstrating prevalence of Ig subclass distribution following CD19 CAR T cell treatment and the probability of generation of BCRs as calculated by the OLGA algorithm. (**B**) Comparison of expanded clones based on VDJ gene annotation before and after CD19 CAR T cell treatment. (**C**) Top 10 expanded clone analysis on their sharedness and repertoire proportion. Statistical analyses were performed using the median and *t* test with Benjamini-Hochberg correction. Significant differences are indicated as follows: ****P ≤ 0.0001.

In accordance with the shift in B cell subpopulations and the reduction of memory B cells, we observed a virtual disappearance of IgG- and IgA- expressing B cells, while IgM- and IgD-expressing B cell clones expanded (Figure 4A); this expansion was in accordance with our previously published data (14, 16). B cells that repopulated after CD19 CAR T cell treatment additionally displayed an increase in the generation probability of their currently expressed immunoglobulin chains, in line with a naive and unexperienced repertoire and consistent with a reset of the B cell memory compartment (Figure 4A).

We additionally performed a characterization of B cell clones that displayed an expansion prior to and after CD19 CAR T cell treatment, respectively. Here we identified several expanded IgG- and IgA-expressing clones that disappeared in response to CD19 CAR T cells, whereas clones that expanded after therapy exclusively showed expression of IgM or IgD (Figure 4, B and C).

## Discussion

SLE represents a heterogenous spectrum of systemic autoimmune diseases that share common denominators. Apart from SLE-typical clinical features, patients with SLE display an increased activation and autoreactivity within the B cell and plasma cell compartments (3), the presence of antinuclear antibodies (2), an elevated type I IFN signature (5). Although these findings have resulted in the development of various targeted therapeutic approaches such as depletion of B cells or blockade of IFN signaling, we lack a full understanding of the molecular base of this disease and of the series of events that trigger the observed break in self-tolerance or onset of SLE (17). Targeted therapies such as monoclonal antibodies and CAR T cells not only offer promising therapeutic tools but simultaneously allow a reverse translational approach that enables us to better understand the underlying disease itself and thereby identify novel therapeutic targets and treatment strategies.

Our current data show that a CD19 CAR T cell–based therapy serves as effective strategy to selectively reset the B cell compartment without affecting the composition of other immune cell subsets or the nature of the T cell response. The overall composition of monocytes, DCs, and T cell subsets as well as the TCR repertoire remained largely unaltered, whereas we observed profound changes in the distribution of B cell subsets, the B cell receptor repertoire, and the B cell–dependent immune memory in response to this treatment strategy. Formally, we cannot exclude that the lymphotoxic conditioning therapy with fludarabine and cyclophosphamide, which is given prior to CAR T cell therapy, contributes to the beneficial effects observed in this cohort of patients with SLE. The selective reset of the B cell response, however, suggests that the therapeutic effect is primarily linked to the action of CD19 CAR T cells.

Notably, our data show that CD19 CAR T cell therapy is highly effective in eliminating IgG^+^ and IgA^+^ memory B cells, which fail to repopulate the peripheral blood despite the reappearance of other B cell subsets following CD19 CAR T cell treatment. This finding provides a potential explanation for the clinical efficacy of this therapeutic approach, as persistence and/or reappearance of memory B cells upon CD20 antibody–mediated B cell depletion have been previously associated with treatment failures and disease relapses (18, 19). In the future, it will be important to directly assess potential differences in the molecular signature of repopulating B cells in response to different B cell–depleting strategies such as CD19 CAR T cells and CD20 antibodies, respectively.

The major CD19 CAR T cell–induced change observed in PBMCs other than B cells was a profound reduction in the type I IFN signature in monocytes and T cells. This finding clearly indicates that the aberrant B cell response in SLE (directly or indirectly) triggers the enhanced IFN signature observed in PBMCs of patients with SLE and suggests that the increase in type I IFN signaling is a consequence rather than a cause of the increased activation of autoreactive B cells in SLE. Notably, we did not observe changes in the expression of type I IFNs in B cells of patients with SLE nor a difference in IFN expression in B cells or other PBMC subsets prior to and after CD19 CAR T cell therapy (data not shown). These observations suggest that, although B cells are (direct or indirect) triggers of the IFN signature in PBMCs, they do not act as the direct source of type I IFNs themselves. A likely explanation is that B cell–derived autoantibodies and/or autoantibody-containing immune complexes trigger IFN expression in tissue-resident immune cells, which consequently provokes the IFN response observed in PBMCs of patients with SLE. Candidate cells that produce type I IFNs in SLE include different DC subsets such as plasmacytoid DCs as well as tissue macrophages that can produce large amounts of type I IFNs in response to toll like receptor activation triggered by immune complexes that contain endogenous nucleic acids (20).

Our current data help to shed light on both the role of B cells as well as on the cascade of events that results from B cell activation and autoreactivity. They additionally provide a rationale to potentially combine and arrange different synergistic preventive and therapeutic approaches, such as blocking of IFN signaling and B cell depletion in order to allow an efficient and rapid suppression of inflammation and enabling a reset autoimmunity.

## Methods

### Sex as biological variant.

This study included both male and female patients (Supplemental Table 1). Although the incidence of SLE is increased in females in comparison with males, we do not consider sex as major biologic variable in terms of the response to CD19 CAR T cell therapy.

### Study participant details.

Peripheral blood samples were collected from donors before CD19 CAR T cell therapy as well as after repopulation of B cells after obtaining informed consent in accordance with the CARE guidelines and approval from the IRB of the University Clinic of Erlangen under the license 334_18 B. The donor demographic information is provided in Supplemental Table 1. PBMCs were isolated by using SepMate-50 (Stemcell Technologies) and Ficoll-Paque gradient centrifugation (1200 x g for 10 minutes at romm temperatures) according to manufactures protocol. The isolated PBMCs were then washed and resuspended in PBS with 10 mM EDTA (Invitrogen) for scRNA-Seq and in MACS buffer (PBS, 2% FCS, 10 mM) for downstream magnetic B cell isolation. B cell isolation was performed using EasySep Human B Cell Enrichment Kit (Stemcell Technologies) according manufacture protocol.

### Library preparation for scRNA-Seq and repertoire sequencing.

For scRNA-Seq and immune repertoire analysis, we employed the Chromium Next GEM Single cell 5′Reagent Kits v2 (10X Genomics) to generate single-cell cDNA libraries from isolated PBMCs. To increase the yield for B cells we performed cDNA library generation on isolated B cells. The isolated PBMCs and B cells were encapsulated into droplets, and reverse transcription of RNA was performed to generate barcoded cDNA molecules. The final libraries were then sequenced on an Illumina NovaSeq sequencer with 150 bp long paired-end reads. B cell receptor (BCR) and T cell receptor (TCR) regions were amplified through PCR using locus-specific primers targeting constant regions of the respective TCR or BCR. The amplification products were then subjected to high-throughput sequencing on an Illumina NovaSeq platform with 150 base pair long paired-end reads aiming at a mean of 15,000 reads per cell. Reads were converted to FASTQ format using mkfastq from Cell Ranger 7.0.0 (10X Genomics).

### scRNA-Seq and repertoire data analysis.

The raw scRNA-Seq data were preprocessed and analyzed using the Cell Ranger multi pipeline (v.7.0.0). The reads were mapped to the human reference genome (GRCh38-2020-A). Cell filtering, barcode counting, and unique molecular identifier (UMI) counting and downstream data handling were performed using the Seurat R package (v.4.3) to obtain the count matrices for each individual cell. To filter out low-quality cells, we applied appropriate thresholds for UMIs, number of detected genes, and number of UMIs assigned to mitochondrial genes. All single-patient data sets were integrated into 1 object for global analysis. We performed dimensionality reduction using principal component analysis (PCA) and cell clustering. The number of PCs to be used for clustering was identified using the ElbowPlot function included in the Seurat R Package, and clustering resolution was decided on with the clustree R package (v.0.5.0). Cluster identity was determined using the EnrichR R package (v3.2) as well as common marker expression. T cells and B cells were identified using common marker expression of CD3, CD4, CD8, and CD19. Subsequently T cells and B cells were filtered, selected for cells that have a sequenced TCR or BCR, and reclustered separately (21).

### BCR and TCR sequencing.

Clonotype calling was performed using the Cell Ranger multi pipeline (v.7.0.0). Integration of the BCR/TCR data was done with the Seurat R package (v.4.3), and TCR/BCR diversity and clonality analysis was performed using the scRepertoire R package (v.1.10.0)

### Differential expression and pathway analysis.

Differentially expressed genes (DEGs) for all sets of cells were determined using the FindAllMarkers function of the Seurat package using the default settings. The identified DEGs were then used as input for PEA with the pathfinder R package (v.1.6.4). The Reactome pathway database was used as a reference for pathway specific gene sets (https://www.kegg.jp/kegg/).

### CDR3 region generation probability estimation.

To estimate the likelihood of BCR and TCR CDR3 sequence generation, we utilized the Optimized Likelihood estimate of immunoGlobulin Amino-acid sequences (OLGA) tool (v.1.2.4). Cells that are not compatible with any recombination scenario were filtered out.

### Statistics.

Statistical analysis was carried out in R, and a complete description is available in the associated figure legends. Median values and 1 tailed *t* test with Benjamini-Hochberg correction, was used for statistical analysis for OLGA analysis. For generating figures, we used the R packages ggplot2 (v.3.4.2), scRepertoire (v1.7.2), EnhancedVolcano (v.1.14.0), ComplexHeatmap (v.2.16.0), and pheatmap (v.1.0.12) as well as Adobe Illustrator CC 2018 (v.22.1).

### Study approval.

The study has been approved by the ethics committee of the University Hospital Erlangen, Germany.

### Data availability.

The scRNA-Seq and repertoire data set generated during this study is available at GEO Platform (accession no. GSE263931). Supporting data values associated with the main manuscript and supplement material are available in the Supporting Data Values file.

## Author contributions

GK, GS, DM, and AM designed the study and wrote the manuscript. AW, DC, and TW designed the study, analyzed data, and performed experiments. FM, AB, an RGB wrote the manuscript and provided input.

## Supplementary Material

Supplemental data

Supporting data values

## Version 1 05/09/2024

In-Press Preview

## Version 2 06/24/2024

Electronic publication
