# Supplementary material for: Selective CAR T cell–mediated B cell depletion suppresses IFN signature in SLE
Source: JCI Insight. 2024 May 9;9(12):e179433. doi: 10.1172/jci.insight.179433 (PMC11383166; doi:10.1172/jci.insight.179433)
Supplement: Supplemental data [file jciinsight-9-179433-s120.docx]

| **ID** | **Sex** | **Age*** | **Disease** |
| --- | --- | --- | --- |
| SLE1 | Female | 20 | SLE |
| SLE2 | Male | 22 | SLE |
| SLE3 | Female | 22 | SLE |
| SLE4 | Female | 24 | SLE/nephrotic snydrom |
| SLE5 | Female | 18 | SLE |
| SLE6 | Female | 38 | SLE |
| SLE7 | Female | 33 | SLE |

* at time of CD19 CAR T cell application

**Table S1. Summary of patient information treated with CD19 CAR T cells. Sample identifiers related to Figures 1-3.**

| **ID** | **CAR T cell application** | **Time point pre therapy** | **B cells/µL** | **Time point post therapy** | **B cells/µL** |
| --- | --- | --- | --- | --- | --- |
| SLE1 | 22.03.2021 | 17.03.2021 | 19 | 21.10.2021 | 148 |
| SLE2 | 09.08.2021 | 04.08.2021 | 85 | 30.03.2022 | 108 |
| SLE3 | 13.12.2021 | 23.11.2021 | 177 | 11.05.2021 | 23 |
| SLE4 | 20.12.2021 | 06.12.2021 | 280 | 11.05.2021 | 853 |
| SLE5 | 07.02.2022 | 25.01.2022 | 234 | 08.06.2022 | 15 |
| SLE6 | 09.05.2022 | 25.04.2022 | 1 | 06.12.2022 | 93 |
| SLE7 | 26.09.2022 | 20.09.2022 | 25 | 05.12.2022 | 282 |

**Table S2. Summary of time points for the effect CD19 CAR T cells treatment analysis. Sample identifiers related to Figures 1-3.**
